# Standardized warfarin monitoring decreases adverse drug reactions

**DOI:** 10.1186/s12875-019-1041-5

**Published:** 2019-11-07

**Authors:** Lisa B. E. Shields, Paula Fowler, Diane M. Siemens, Douglas J. Lorenz, Kenneth C. Wilson, Steven T. Hester, Joshua T. Honaker

**Affiliations:** 1 Norton Neuroscience Institute, Norton Healthcare, 210 East Gray Street, Suite 1102, Louisville, KY 40202 USA; 2 Norton Medical Group, Norton Healthcare, 4801 Olympia Park Plaza, Suite 3000, Louisville, KY 40241 USA; 3 Department of Bioinformatics & Biostatistics, University of Louisville, Louisville, KY 40292 USA

**Keywords:** Family practice, Primary care provider, Cardiologist, Warfarin, Adverse drug reaction

## Abstract

**Background:**

While warfarin is the most commonly prescribed medication to prevent thromboembolic disorders, the risk of adverse drug reactions (ADR) poses a serious concern. This prospective study evaluated how primary care providers (PCP) and cardiologists at our Institution managed patients treated with warfarin with the goal of decreasing the number of warfarin ADRs.

**Methods:**

A multidisciplinary anticoagulation task force was established at our Institution in 2014 to standardize warfarin monitoring and management. Between 2013 and 2017, we analyzed patients who were prescribed warfarin by their PCP or cardiologist upon hospital discharge and in the ambulatory setting to determine the international normalized ratio (INR) within 5, 10, and 30 days after discharge, time in therapeutic range (TTR), number of severe warfarin ADRs, and total and average cost reduction of all severe warfarin ADRs to determine whether there was an organizational cost savings following the implementation of standardized warfarin care.

**Results:**

The warfarin ADR rate significantly decreased over the 5-year period, from 3.8 to 0.98% (*p* < 0.0001). The proportion of warfarin prescriptions out of all anticoagulants significantly decreased, from 72.2 to 42.1% (*p* < 0.001). The proportion of individuals who received an INR at 5, 10, and 30 days after hospital discharge compared to the total number of patients prescribed warfarin significantly increased (*p* < 0.001). The total cost of severe warfarin ADRs decreased by 57.6% between 2013 and 2017.

**Conclusions:**

This study serves as a model to reduce the number of severe warfarin ADRs by the following tactics: (1) educating PCPs and cardiologists about evidence-based guidelines for warfarin management, (2) increasing the use of our Institution’s electronic warfarin module, and (3) enhancing patient compliance with obtaining INR.

## Background

The epidemiology of anticoagulant prescriptions has been rapidly changing over the past decade. Warfarin, a vitamin K antagonist, has historically been the most frequently used oral anticoagulant in the world for patients with venous thrombosis, pulmonary embolism, atrial fibrillation, prosthetic heart valves, recurrent myocardial infarction, and stroke [1–7]. Thromboembolic disorders significantly contribute to morbidity and mortality. The direct oral anticoagulants (DOACs) have a lower incidence of major bleeding than warfarin, minor drug and food interactions, rapid onset and offset, short half-life, and stable pharmacokinetics eliminating the need for regular monitoring and dose adjustment [8, 9]. It has been reported that the prevalence of warfarin use decreaseed from 69.8% in 2008 to 42.2% in 2014 paralleled with an increase in the DOAC rivaroxaban from 1.3% in 2010 to 12.1% in 2011 [1].

The decision whether to initiate warfarin therapy should weigh the potential risks (hemorrhage, drug interactions, and routine monitoring requirements) and benefits (prevention of thromboembolic event) on an individual basis [10]. According to the Stroke Prevention in Atrial Fibrillation trial, the rate of major bleeding for patients treated with warfarin is 2.3% per year [11]. The incidence of hemorrhage in patients treated with warfarin is directly related to the adequacy of control [5]. The generic prothrombin time or protime (PT) historically has been used to monitor warfarin levels. To ensure a consistent and standardized model for PT, the World Health Organization in 1982 developed the international normalized ratio (INR) [12–14]. INR monitoring is performed often when warfarin therapy is initiated until a level in the optimal therapeutic range is attained [3, 15–17]. Close monitoring of warfarin is integral to the management of patients to maintain anticoagulation within the therapeutic window [18].

The Rosendaal method is commonly used to determine time in therapeutic range (TTR), specifically, an INR between 2.0 and 3.0 [19]. This method calculates the percentage of time a patient’s INR is within the desired therapeutic range as well as INR-specific incidence rates of either thromboembolic or hemorrhagic events. It also determines the optimal pharmacological effects of anticoagulation and monitors both the outcomes and compliance of patients.

An anticoagulation task force was established at our Institution in 2014 aimed at educating PCPs and cardiologists about evidence-based guidelines for warfarin management, increasing the use of our Institution’s electronic warfarin module, and enhancing patient compliance with obtaining INRs. The primary objective of our study was to decrease the number of severe warfarin ADRs. Additional objectives included whether the establishment of the anticoagulation task force had a positive effect on TTR and severe ADRs. We hypothesized that the numerous strategies established by the anticoagulation task force such as providers using the electronic warfarin module and patients’ obtaining timely INRs and follow-up visits with their provider would positively impact our leading objective of reducing warfarin ADRs.

In the current study, we present our findings of the number of patients prescribed warfarin at hospital discharge and who obtained an INR within 5, 10, and 30 days of hospital discharge, the TTR, the number of patients treated with warfarin compared to other anticoagulant medications, the use of the electronic health module by PCPs and cardiologists, and the number and cost change of severe warfarin ADR events.

## Methods

Under an institutional review board-approved protocol, our prospective study (January 1, 2013- December 31, 2017) investigated the number of severe warfarin ADRs after hospital discharge and the management of patients prescribed warfarin by approximately 200 PCPs and 48 cardiologists in an ambulatory setting at our Institution. The study design was a trend analysis in which the data were derived from a comprehensive records review involving all prescriptions of anticoagulants at our Institution over this 5-year time period. Prior to the initiation of this study, our Institution observed that warfarin was the leading medication associated with ADRs. Our Institution defined a severe ADR as one in which the patient outcome was death, life-threatening (real risk of dying), hospitalization (initial or prolonged), disability (significant, persistent, or permanent), congenital anomaly, or requiring intervention to prevent permanent impairment or damage. Patients who were solely evaluated in the Emergency Department (ED) and not admitted to the hospital did not experience a severe ADR. Established at our Institution in 2014, the anticoagulation task force aimed to improve adherence of evidence-based management guidelines established by the American College of Cardiology and to standardize monitoring and management of warfarin by implementing defined dosing algorithms and regulating treatment strategies with INRs with the goal of decreasing the number of warfarin ADRs (Table 1). The task force consisted of the following disciplines: ambulatory adult PCP, cardiology, adult hospitalists, clinical effectiveness engineers, and information technology, pharmacy, hospital, and ambulatory operations.

**Table 1:** Goals of the anticoagulation task force at our Institution

Decrease the number of severe warfarin ADRs	
Educate PCPs about the electronic warfarin module in Epic	
Standardize monitoring and management of warfarin by improving adherence of evidence-based guidelines advocated by the American College of Cardiology	
Implement defined warfarin dosing algorithms	
Enhance patient compliance with obtaining INRs to ensure that they remain in the TTR	
Decrease costs related to warfarin-related readmissions	

*ADR* Adverse drug reaction, *PCP* Primary care provider, *INR* International normalized ratio, *TTR* Time in therapeutic range

The warfarin data were evaluated over 5 years: (1) baseline - prior to the anticoagulation task force (1/1/2013–12/31/2013) and (2) 1–4 years later (1/1/2014–12/231/2017). Numerous measures were implemented at our Institution following the initiation of the task force (Table 2). The electronic warfarin module is a template build software that utilizes the electronic medical record software application Epic which may be accessed by treating physicians at all times. Prior to anticoagulation task force, providers monitored their patients’ INRs without the use of the warfarin module in Epic. The electronic warfarin module adds and archives lab results, displays data trends, and offers standardized recommendations and guidelines regarding the appropriate management of warfarin. By educating providers about the warfarin module and incorporating this module in their daily practice, it was easier and more reliable to monitor INRs. Additionally, providers were able to observe trends in relation to the module usage, TTRs, and patients treated with warfarin. Special attention focused on dramatic changes in a patient’s INR results, necessitating more frequent PCP visits. Following the initiation of the task force, PCPs were educated about numerous topics, including (1) a video and link informing PCPs about how to use the electronic warfarin module, specifically, how to monitor their patients’ INRs to ensure that they remain in the TTR, (2) a hyperlink on Epic indicating the appropriate warfarin dosing protocols as established by the American College of Cardiology, and (3) how to educate their patients about warfarin use, particularly how to contact their PCPs for the medication. Furthermore, a visual calendar and after-visit summary permit patients to view their previous INR values, upcoming appointments, and warfarin dosing.

**Table 2:** Measures implemented at our Institution following the initiation of the anticoagulation task force to standardize warfarin monitoring and management

The electronic warfarin module in Epic at our Institution adds and archives lab results, displays data trends, and offers standardized recommendations and guidelines regarding the appropriate management of warfarin	
Educational video and link inform PCPs about how to use the electronic warfarin module, specifically, how to monitor their patients’ INRs to remain in the TTR	
Hyperlink in Epic indicates appropriate warfarin dosing protocols as established by the American College of Cardiology	
PCPs learn how to educate patients about warfarin use	
BPAs and a visual calendar regarding future visit follow-ups, timely lab draws, and patient education appear in the electronic warfarin module in the electronic medical record when a provider orders warfarin	
A visual calendar and after-visit summary permit patients to view their previous INR values, upcoming appointments, and warfarin dosing	

*PCP* Primary care providers, *INR* International normalized ratio, *TTR* Time in therapeutic range, *BPA* Best practice alert

A modified Rosendaal method was used to determine the TTR [19]. This method calculates a patient’s TTR by incorporating the frequency of INR measurements and the corresponding INR values against the patient’s INR goals. The Rosendaal method assumes that changes between consecutive INR measurements are linear over time.

The total and average cost reduction of all severe warfarin ADRs was calculated to determine whether there was an organizational cost savings following the implementation of standardized warfarin care. The average cost was per patient discharged with warfarin who experienced a severe warfarin ADR. Our Institution does not provide the actual costs of severe warfarin ADRs and, therefore, we present the cost savings as a percentage change.

### Statistical analysis

A chi-square test for trend was utilized to evaluate for all trends in proportions over time except for trend for TTR which used the Poisson generalized linear model. The change in the distribution of type of provider (PCP, cardiologist, or other medical specialties) over time was evaluated with a chi-square test. The change in the number of warfarin prescriptions over time was evaluated with the Mann-Kendall test for trend. A *p* < 0.05 indicated statistical significance.

## Results

### Warfarin prescribed at hospital discharge

The number of patients prescribed warfarin at hospital discharge decreased over our 5-year study, from a total of 925 at baseline in 2013 to 665 in 2017 (Table 3). The majority of patients were given warfarin by their PCP. Other medical specialties that prescribed warfarin at hospital discharge include neurosurgery, spinal surgery, general surgery, hematology, oncology, and gastroenterology. The distribution of the types of physicians (PCPs, cardiologist, other specialties) prescribing warfarin was significantly different over the 5 years (*p* < 0.001), however, there was no discernible trend over time in this distribution.

**Table 3:** Warfarin module metrics in the electronic medical record at our Institution

Metric	2013 (Baseline)	2014	2015	2016	2017	*P*-value
Warfarin prescribed at hospital discharge	*N* = 925	*N* = 1027	*N* = 954	*N* = 740	*N* = 665	*p* < 0.001
PCP	753 (81.4%)	878 (85.5%)	735 (77.0%)	617 (83.4%)	487 (73.2%)	
Cardiology	62 (6.7%)	38 (3.7%)	20 (2.1%)	24 (3.2%)	59 (8.87%)	
Others	110 (11.9%)	111 (10.8%)	199 (20.9%)	99 (13.4%)	108 (16.2%)	
Number of patients on warfarin compared to other anticoagulants	1658/2295 (72.2%)	2073/3430 (60.4%)	2294/4451 (51.5%)	2247/5135 (43.8%)	2650/6301 (42.1%)	*p* < 0.001
INR within 5 days of hospital discharge	*N* = 308 (33.3%)	*N* = 376 (36.6%)	*N* = 353 (37.0%)	*N* = 309 (41.8%)	*N* = 293 (44.0%)	*p* < 0.01
PCP	250 (33.2%)	316 (36.0%)	297 (39.4%)	253 (41.0%)	218 (44.8%)	
Cardiology	14 (22.6%)	15 (39.5%)	7 (35.0%)	12 (50.0%)	32 (54.2%)	
Others	44 (40.0%)	45 (40.5%)	49 (24.6%)	44 (44.4%)	40 (37.0%)	
INR within 10 days of hospital discharge	*N* = 456 (49.3%)	*N* = 573 (55.8%)	*N* = 510 (53.5%)	*N* = 460 (62.2%)	*N* = 397 (59.7%)	*p* < 0.01
PCP	369 (49.0%)	487 (55.5%)	409 (55.6%)	382 (61.9%)	299 (61.4%)	
Cardiology	28 (45.0%)	25 (65.8%)	13 (65.0%)	15 (62.5%)	37 (62.7%)	
Others	59 (53.6%)	61 (55.0%)	88 (44.2%)	63 (63.6%)	54 (50.0%)	
INR within 30 days of hospital discharge	*N* = 684 (73.9%)	*N* = 792 (77.1%)	*N* = 742 (77.8%)	*N* = 622 (84.1%)	*N* = 537 (80.8%)	*p* < 0.01
PCP	550 (73.0%)	680 (77.4%)	586 (77.8%)	516 (83.6%)	402 (82.5%)	
Cardiology	51 (82.2%)	33 (86.8%)	19 (95.0%)	22 (91.7%)	49 (83%)	
Others	83 (75.4%)	79 (71.2%)	137 (68.8%)	84 (84.8%)	78 (72.2%)	
Time in therapeutic range						
PCP	43,832/71,756 (61.1%)	65,727/108,481 (60.6%)	71,658/116,881 (61.3%)	74,025/121,448 (61.0%)	67,773/108,414 (62.5%)	*p* = 0.191
Cardiology	452,620/658,264 (68.8%)	439,047/643,609 (68.2%)	458,181/670,446 (68.3%)	444,692/646,642 (68.8%)	431,225/626,779 (68.8%)	*p* = 0.182
Electronic health module usage						
PCP	6657/9657 (68.9%)	8356/11,438 (73.0%)	9792/12,680 (77.2%)	10,608/12,832 (82.7%)	10,269/12,202 (84.2%)	*p* < 0.001
Cardiology	5154/6733 (76.5%)	7086/9327 (76.0%)	8748/10,844 (80.7%)	8737/10,338 (84.5%)	8127/9520 (85.4%)	*p* < 0.001

*PCP* Primary care provider, *INR* International normalized ratio, *ADR* Adverse drug reaction

### Prescriptions of warfarin compared to other anticoagulants

The proportion of warfarin prescriptions out all anticoagulants decreased over the 5 years, from 72.2 to 42.1% (*p* < 0.001) (Table 3).

### INR within 5, 10, and 30 days of hospital discharge and time in therapeutic range

The proportion of patients who had an INR within 5, 10, and 30 days of hospital discharge out of the total number of patients who were prescribed warfarin by all PCPs, cardiologists, and other specialties significantly increased over the 5-year period (*p* < 0.01 for each time period) (Table 3). At 5-days post-discharge, the proportion also significantly increased for PCPs and cardiology (*p* < 0.001 for both). The proportion significantly increased for PCPs at 10- and 30-days post-discharge (*p* < 0.001 for both).

### Electronic warfarin module usage and time in therapeutic range

Use of the electronic health module significantly increased between baseline in 2013 and 2017 for both PCPs (68.9 to 84.2%, *p* < 0.001) and cardiologists (76.5 to 85.4%, *p* < 0.001). The TTR is represented by a ratio of the number of days the INR was in the therapeutic range for all patients treated with warfarin to the total number of days all patients were treated with warfarin. The TTR was significantly higher for cardiologists compared to PCPs for all 5 years of our study (*p* < 0.0001).

### Severe adverse drug reactions attributed to warfarin

The number of severe ADRs due to warfarin decreased at our Institution between 2013 and 2017, from 63 to 26 (Table 4). The warfarin ADR rate reflects the ratio of the number of severe warfarin ADRs to the total number of warfarin prescriptions. The warfarin ADR rate significantly decreased over the 5-year period of this study, from 3.8% at baseline in 2013 to 0.98% in 2017 (*p* < 0.0001; Table 4).

**Table 4:** Adverse drug reactions attributed to warfarin at our Institution

Metric	2013 (Baseline)	2014	2015	2016	2017	*P*-value
Number of severe warfarin ADRs	63/1658	37/2073	54/2294	28/2247	26/2650	
Warfarin ADR rate	3.80%	1.78%	2.35%	1.25%	0.98%	*p* < 0.0001

*ADR* Adverse drug reaction, Severe ADR: death, life-threatening (real risk of dying), hospitalization (initial or prolonged), disability (significant, persistent, or permanent), congenital anomaly, or requiring intervention to prevent permanent impairment or damage

### Total cost change of severe warfarin ADRs

Between 2013 and 2017, the total and average cost of all severe warfarin ADRs decreased by 57.6 and 20.3%, respectively.

## Discussion

The goal of warfarin therapy is to prescribe the lowest dose necessary to prevent clot formation or expansion while averting the ADR associated with over-anticoagulation [4]. In this study, we present three strategies to reduce the number of severe warfarin ADRs and cost: (1) educating PCPs and cardiologists about evidence-based management guidelines established by the American College of Cardiology for warfarin management, (2) increasing the use of the warfarin module by PCPs and cardiologists, and (3) enhancing patient compliance with obtaining INR after hospital discharge.

### Time in therapeutic range and INR

TTR is an important measure to assess the safety and efficacy of warfarin treatment [20–23]. Maximizing TTR, specifically maintaining an INR between 2 and 3, plays the most critical role in preventing stroke, major hemorrhage, or death [24]. The average TTR has ranged from 56% in retrospective studies to 65% in randomized controlled trials [21, 23]. Similar to the present study, Pokorney and colleagues used a modified Rosendaal method to determine the TTR of patients with atrial fibrillation on warfarin [21]. Patients with the highest risk of bleeding and stroke had the lowest TTRs.

In our current study, the proportion of patients who had an INR within 5, 10, and 30 days of hospital discharge out the total number of patients who were prescribed warfarin by all PCPs, cardiologists, and other specialties significantly increased over the 5-year period (*p* < 0.01 for each time period). Interestingly, the proportion significantly increased for PCPs at 5, 10, and 30 days post-discharge (*p* < 0.001 for each time period).

### Adverse drug reaction involving warfarin

The management of ADRs in the United States costs approximately $30.1 billion annually due to the increased number of hospital visits and prolonged hospital stay [25]. Patient non-compliance may lead to an ADR causing negative clinical and economic consequences, including a reduced quality of life [26]. Warfarin is one of the most commonly prescribed medications that cause drug-related adverse events leading to ED visits [27, 28].

While predictors of warfarin-associated adverse events have been reported in hospitalized patients [29–32], a paucity of studies addressed attempts to reduce warfarin ADRs in an outpatient setting [33, 34]. Salinero and Hyman reported a model of reducing warfarin ADRs with a nurse led anticoagulation clinic in South Miami, Florida [34]. An interdisciplinary team with physicians, nurses, and nurse practitioners was established in response to the increased number of hospital admissions for warfarin toxicity. Physicians referred their warfarin patients to the anticoagulation clinic with evidence-based clinical warfarin management, replete with dosing adjustments and follow-up intervals. Following the implementation of the anticoagulation clinic, the number of hospital admissions related to warfarin ADRs decreased from 27 in 2006 to 9 in 2015. The goal of reducing warfarin ADRs was accomplished in both Salinero and Hyman’s work and the present study, specifically, through an anticoagulation clinic in the former and an electronic warfarin module in ours.

Utilizing lab-based patient-specific INR and pharmacy-based patient specific Vitamin K triggers, Lederer and Best investigated warfarin adverse drug events (ADEs) in both inpatient and outpatient settings of Novant Health System [33]. They developed process improvement and medication management protocols for patients treated with warfarin. The two trigger interventions included (1) the laboratory identified all patients with an INR > 3 and (2) that pharmacy identified all patients who received Vitamin K as a warfarin reversal agent. Triggered charts underwent harm classification assignments based on the National Coordinating Council for Medication Error Reporting and Prevention Index. These authors reported reductions in warfarin ADEs for both inpatients (45%) and outpatients (52%) [33].

The present study closely reflects the goals and findings of Lederer and Best. Physician education as to the potential problems associated with warfarin management was the primary intervention in both studies. Following the multidisciplinary meeting in 2014, PCPs and cardiologists at our Institution were taught how to use the warfarin electronic module to monitor their patients treated with warfarin. Our results confirmed that use of the electronic health module significantly increased between baseline in 2013 and 2017 for both PCPs (68.9 to 84.2%, *p* < 0.001) and cardiologists (76.5 to 85.4%, *p* < 0.001). Over this 5-year period, the number of patients prescribed warfarin at hospital discharge decreased, from a total of 925 at baseline in 2013 to 665 in 2017. Furthermore, the proportion of warfarin prescriptions out all anticoagulants decreased over the 5 years, from 72.2 to 42.1% (*p* < 0.001). We attribute the decreased percentage to a trend for patients to initiate safer DOACs that did not require retrieval of INRs. The increased number of patients was most likely due to the larger patient population treated at our Institution over the course of our study. Furthermore, a large population of patients continued to consume warfarin primarily due to the lower cost of this medication.

As PCPs and cardiologists used the warfarin module more frequently and were educated about evidence-based guidelines for warfarin monitoring and as more patients obtained their INR after hospital discharge, there was a simultaneous decrease in the number of severe warfarin ADRs and warfarin ADR rate at our Institution. This significant reduction in warfarin ADRs may reflect the consistent management of outliers with the defined algorithms such that their time outside of the TTR was less likely to result in patient harm. The decline in the ADR rate may also be due to noncompliant unstable patients who have discontinued warfarin and initiated DOACs or other anticoagulants. Some patients who experienced a warfarin-related ADR may have been referred to another facility outside of our Institution, and we were unable to capture the number of patients who may have done so. Therefore, these patients would have been missed from our study. In addition, the total and average cost of all severe warfarin ADRs decreased by 57.6 and 20.3%, respectively, between 2013 and 2017.

### Strengths and limitations of the present study

Our 5-year study serves as a unique model for incorporating an electronic warfarin module into clinical family practice with the goal of closely monitoring INR to increase the time in the therapeutic window thereby decreasing warfarin ADRs, ED visits, readmissions, costs, and morbidity and mortality. The initiative presented in this study established standardized warfarin monitoring and management processes at our Institution by disseminating evidence-based guidelines to the PCPs and cardiologists. The robust warfarin module plays a valuable role in many respects, including the ease of tracking INR, offering a hyperlink to the American College of Cardiology guidelines to warfarin therapy, and providing a visual calendar with an after-visit summary to alert patients of their previous INR values, upcoming appointments, and dosing regimen. This model has served to both educate PCPs about appropriate dosing and monitoring of warfarin levels and increase patient compliance with INR visits.

As our warfarin module in the electronic medical record is in its early stages at our Institution, both the average TTR and the number of patients obtaining INR at 5, 10, and 30 days after being prescribed warfarin at hospital discharge should dramatically rise with continued use of the warfarin module. As the patient population treated with warfarin in this study was not a static group, volatility in the TTR until it was stabilized in new patients may play a role in the TTR remaining in the low 60% for the 5 years of this study. Additionally, some of the statistical tests, in particular those for cardiology and the other medical specialties, may not have been sufficiently powered to detect trends due to the smaller number of patients.

As the objective of our study was to observe the changes in relation to the implementation of the various measures aimed at decreasing warfarin ADRs, we did not have a control group. While the lack of a control group was a limitation in the present study, we believed that our efforts would be valuable for the entire population. Therefore, we wanted to implement the constructive strategies throughout our Institution for all patients treated with warfarin instead of withholding positive modifications from certain members of our community. Thus, we are unable to demonstrate the differences between the group which benefitted from alterations incorporated by the anticoagulation task force and a control group.

Further intervention is warranted to teach PCPs at their respective clinics to use the warfarin module, to ensure appointments for INR testing are made at the time of hospital discharge, and to arrange consultations with a nurse navigator for patients who are discharged with a history of severe warfarin ADRs. Continued observation of the various metrics in the warfarin module in the EMR is necessary to incorporate revisions. We aim to establish a staffing unit at our hospital which will ensure that patients are discharged with a filled bottle of warfarin, a warfarin prescription, and their subsequent INR schedule. PCPs at our Institution are actively pursuing a standardized and centralized anticoagulation management center model. This site will be directed by dedicated nurse practitioners with pharmacy support to manage warfarin lab values and adjust dosing accordingly.

## Conclusion

Close observation of warfarin levels is imperative to mitigate the potentially devastating sequela of a thromboembolic event. This article serves as a model to reduce the number of severe warfarin ADRs by educating PCPs and cardiologists about evidence-based guidelines for warfarin management and enhancing patient compliance with obtaining INR. Standardization of warfarin monitoring and management plays a crucial role in reducing the number of severe warfarin ADRs in a primary care setting in a metropolitan community.

## Data Availability

All of the raw data presented in this study may be requested from the corresponding author of this work.

## Abbreviations

ADR: Adverse drug reaction
DOAC: Direct oral anticoagulants
INR: International normalized ratio
PCP: Primary care provider
PT: Protime
TTR: Time in therapeutic range

## References

[1] Alalwan AA, Voils SA, Hartzema AG (2017). Trends in utilization of warfarin and direct oral anticoagulants in older adult patients with atrial fibrillation. Am J Health Syst Pharm.

[2] Guyatt GH, Akl EA, Crowther M (2012). Executive summary: antithrombotic therapy and prevention of thrombosis, 9th ed: American College of Chest Physicians evidence-based clinical practice guidelines. Chest.

[3] Jacobs LG (2008). Warfarin pharmacology, clinical management, and evaluation of hemorrhagic risk for the elderly. Cardiol Clin.

[4] Krynetskiy E, McDonnell P (2007). Building individualized medicine: prevention of adverse reactions to warfarin therapy. J Pharmacol Exp Ther.

[5] Ouirke W, Cahill M, Perera K (2007). Warfarin prevalence, indications for use and haemorrhagic events. Ir Med J.

[6] Tadros R, Shakib S (2010). Warfarin--indications, risks and drug interactions. Aust Fam Physician.

[7] Turakhia MP (2011). Quality of stroke prevention care in atrial fibrillation: many moving targets. Circ Cardiovasc Qual Outcomes.

[8] Jun M, Lix LM, Durand M (2017). Comparative safety of direct oral anticoagulants and warfarin in venous thromboembolism: multicentre, population based, observational study. BMJ.

[9] Mekaj YH, Mekaj AY, Duci SB (2015). New oral anticoagulants: their advantages and disadvantages compared with vitamin K antagonists in the prevention and treatment of patients with thromboembolic events. Ther Clin Risk Manag.

[10] Kirley K, Qato DM, Kornfield R (2012). National trends in oral anticoagulant use in the United States, 2007 to 2011. Circ Cardiovasc Qual Outcomes.

[11] Bleeding during antithrombotic therapy in patients with atrial fibrillation. The Stroke Prevention in Atrial Fibrillation Investigators. Arch Intern Med. 1996;156:409–16. 8607726

[12] Hirsh J, Dalen J, Anderson DR (2001). Oral anticoagulants: mechanism of action, clinical effectiveness, and optimal therapeutic range. Chest.

[13] Perrero PP, Willoughby DF, Eggert JA (2004). Warfarin therapy in older adults: managing treatment in the primary care setting. J Gerontol Nurs.

[14] Pineo G, Hull RD (2003). Coumarin therapy in thrombosis. Hematol Oncol Clin North Am.

[15] Erkens PM, ten Cate H, Buller HR (2012). Benchmark for time in therapeutic range in venous thromboembolism: a systematic review and meta-analysis. PLoS One.

[16] Rose AJ, Hylek EM, Ozonoff A (2011). Risk-adjusted percent time in therapeutic range as a quality indicator for outpatient oral anticoagulation: results of the veterans affairs study to improve anticoagulation (VARIA). Circ Cardiovasc Qual Outcomes.

[17] Wigle P, Hein B, Bloomfield HE (2013). Updated guidelines on outpatient anticoagulation. Am Fam Physician.

[18] Pennsylvania Patient Safety Advisory (2008). Anticoagulation management service: safer care, maximizing outcomes. Pa Patient Saf Advis.

[19] Rosendaal FR, Cannegieter SC, van der Meer FJ (1993). A method to determine the optimal intensity of oral anticoagulant therapy. Thromb Haemost.

[20] Huang D, Wong CL, Cheng KW (2018). Impact of provision of time in therapeutic range value on anticoagulation management in atrial fibrillation patients on warfarin. Postgrad Med J.

[21] Pokorney SD, Simon DN, Thomas L (2015). Patients’ time in therapeutic range on warfarin among US patients with atrial fibrillation: results from ORBIT-AF registry. Am Heart J.

[22] Reiffel JA (2017). Time in the therapeutic range for patients taking warfarin in clinical trials: useful, but also misleading, misused, and overinterpreted. Circulation.

[23] Wieloch M, Sjalander A, Frykman V (2011). Anticoagulation control in Sweden: reports of time in therapeutic range, major bleeding, and thrombo-embolic complications from the national quality registry AuriculA. Eur Heart J.

[24] Gateman D, Trojnar ME, Agarwal G (2017). Time in therapeutic range: warfarin anticoagulation for atrial fibrillation in a community-based practice. Can Fam Physician.

[25] Sultana J, Cutroneo P, Trifiro G (2013). Clinical and economic burden of adverse drug reactions. J Pharmacol Pharmacother.

[26] Leporini C, De SG, Russo E (2014). Adherence to therapy and adverse drug reactions: is there a link?. Expert Opin Drug Saf.

[27] Anthony CJ, Karim S, Ckroyd-Stolarz S (2011). Intensity of anticoagulation with warfarin and risk of adverse events in patients presenting to the emergency department. Ann Pharmacother.

[28] Budnitz DS, Shehab N, Kegler SR (2007). Medication use leading to emergency department visits for adverse drug events in older adults. Ann Intern Med.

[29] Caraballo D, Spyropoulos AC, Mahan CE (2013). Identifying, monitoring and reducing preventable major bleeds in the hospital setting. J Thromb Thrombolysis.

[30] Hug BL, Witkowski DJ, Sox CM (2010). Adverse drug event rates in six community hospitals and the potential impact of computerized physician order entry for prevention. J Gen Intern Med.

[31] Metersky ML, Eldridge N, Wang Y (2016). Predictors of warfarin-associated adverse events in hospitalized patients: opportunities to prevent patient harm. J Hosp Med.

[32] Piazza G, Nguyen TN, Cios D (2011). Anticoagulation-associated adverse drug events. Am J Med.

[33] Lederer J, Best D (2005). Reduction in anticoagulation-related adverse drug events using a trigger-based methodology. Jt Comm J Qual Patient Saf.

[34] Salinero M, Hyman T (2017). Reducing warfarin ADR’s with a nurse led anticoagulation clinic: a new model of patient care.

